# The effect of the quality of the national health security systems in 12 countries on the prevalence of suicide crisis syndrome during the COVID-19 pandemic

**DOI:** 10.1192/bjo.2025.58

**Published:** 2025-05-09

**Authors:** Ferdinand Bortenschlager, Maximilian Lutz, Judith Streb, Claudia I. Astudillo-García, Shira Barzilay, Ksenia Chistopolskaya, Elif Çinka, Sergey N. Enikolopov, Muhammad Ishrat Husain, Oskar Kuśmirek, Vikas Menon, Jefté Peper-Nascimento, Megan L. Rogers, Samira S. Valvassori, Fatma Kantaş Yilmaz, Sungeun You, Manuela Dudeck, Igor Galynker

**Affiliations:** Department of Forensic Psychiatry and Psychotherapy, Ulm University, Ulm, Germany; Department of Epidemiology and Psychosocial Research, National Institute of Psychiatry Ramón de la Fuente Muñiz, Mexico City, Mexico; Department of Community Mental Health, University of Haifa, Haifa, Israel; Eramishantsev Moscow City Clinical Hospital, Lomonosov Moscow State University, Moscow, Russia; Faculty of Health Sciences, University of Health Sciences, Istanbul, Turkey; Department of Medical Psychology, Mental Health Research Center, Moscow, Russia; Campbell Family Mental Health Research Institute, Centre for Addiction and Mental Health, Toronto, ON, Canada; Department of Psychiatry, University of Toronto, Toronto, ON, Canada; Institute of Psychology, Polish Academy of Sciences, Warsaw, Poland; Department of Psychiatry, Jawaharlal Institute of Postgraduate Medical Education and Research, Puducherry, India; Translational Psychiatry Laboratory, Graduate Program in Health Sciences, University of Southern Santa Catarina (UNESC), Criciúma, SC, Brazil; Department of Psychology, Texas State University, San Marcos, TX, USA; Department of Psychology, Chungbuk National University, Cheongju, Chungbuk, Republic of Korea; Icahn School of Medicine at Mount Sinai, Mount Sinai Beth Israel, New York, NY, USA

**Keywords:** COVID-19, suicide crisis syndrome, healthcare system, cross-national sample, multilevel modelling

## Abstract

**Background:**

Limited access to health services and overwhelmed healthcare systems created a challenging environment for those in need of mental health support during the COVID-19 pandemic, and the pandemic impacted suicide risk in several ways.

**Aims:**

The present study aimed to analyse how the quality of the health security systems in 12 countries affected suicide crisis syndrome (SCS) during the pandemic. We hypothesised that countries with robust health systems were better able to respond to the increased demand for (mental) health support, resulting in fewer cases of SCS.

**Method:**

From June 2020 to September 2021, 11 848 participants from 12 different countries took part in an online survey. Besides asking about sociodemographic information, the survey assessed the severity of SCS with the Suicide Crisis Inventory (SCI). The Global Health Security Index and the Legatum Prosperity Health Index were used to operationalise the quality of the national health systems. Multilevel analyses were performed to evaluate the impact of health system quality and COVID-19-associated factors on SCI scores.

**Results:**

SCS was more prevalent among participants with COVID-19 symptoms and in countries with high rates of COVID-19-associated deaths. Multilevel analyses revealed a significant interaction effect of COVID-19 symptoms and national health indices. SCS occurred significantly less frequently in participants with COVID-19 symptoms living in countries with good health security systems.

**Conclusions:**

The challenges posed by the pandemic highlight the necessity to promote accessible and affordable health services to mitigate the negative impact of the pandemic on suicidal ideation and behaviour.

The COVID-19 pandemic has had far-reaching effects on mental health globally.^1–4^ Factors such as social isolation, financial strain, job loss and fear of infection have all contributed to increased stress, depression and anxiety among individuals. Numerous experts have expressed concerns that these stressors, along with limited access to healthcare services during lockdowns and overwhelmed healthcare systems, have created a challenging environment for those in need of support. This may contribute to an increase in suicidal behaviours.^4–7^ Similarly, a review of past epidemics and pandemics reveals a consistent link to rising suicide rates.^8–10^

However, a study analysing suicide rates in 33 countries during the COVID-19 pandemic compared to the pre-pandemic period found no evidence of an increase in suicide rates in the majority of countries.^11^ This finding was confirmed by a living systematic review of 78 articles that investigated the impact of the COVID-19 pandemic on self-harm and suicidal behaviour.^12^ Despite a lack of evidence for a general increase in suicide ideation or behaviour during the pandemic, studies identified subgroups of people who may have shown such increase, such as young people, especially young adults,^13,14^ those with COVID-19 and participants from countries with a higher democracy index.

In general, scholars and experts suggested that health security systems had a clear impact on mental health outcomes during the COVID-19 pandemic.^15,16^ For example, countries with robust health systems, including accessible and well-funded services, were expected to be better able to respond to the increased demand for health support. These systems were able to provide timely interventions, including telehealth services and crisis hotlines, to individuals in distress. On the other hand, countries with limited health infrastructure and resources could be expected to face significant challenges in meeting the escalating needs of their populations, resulting in potential gaps in care and support. Taken together, research shows that healthcare systems differed in terms of the available resources during the COVID-19 pandemic. In line with these findings, political choices regarding interventions for dealing with the challenges of the pandemic varied between countries.^17^

## The present study

The aim of the present study was to analyse the impact of different health security systems on suicide risk during the COVID-19 pandemic. The Global Health Security Index (GHS-I) and the Legatum Prosperity Health Index (LPH-I) were used to operationalise health system quality. The GHS-I summarises countries’ indicators and capabilities, which correspond to a broad concept of health security, including countries’ ability to fight infectious disease outbreaks.^18^ A suitable predictor to capture short-term suicide risk is suicide crisis syndrome (SCS), an acute mental state that typically precedes a suicide (attempt). SCS describes a combination of aversive symptoms, such as feelings of entrapment/frantic hopelessness, intense emotional pain, feelings of panic/dissociation, excessive rumination and fear of dying, in terms of loss of control over death.^19^ To study the syndrome, the Suicide Crisis Inventory (SCI) was developed with the aim to improve the predication of suicidal behaviour by asking indirectly about SCS-associated symptoms. SCS is distinct from suicidal behaviour and suicidal ideation (or thoughts). Suicidal behaviour refers to any action taken with the intent to end one’s life, including suicide attempts and preparatory behaviours. Suicidal ideation refers to persistent thoughts about suicide, which may not always be linked to a specific crisis. These thoughts can range from vague, passive feelings of wanting to die or not wanting to live, to more active and detailed plans for suicide. Suicidal ideation can occur in a chronic or long-term context and may fluctuate in severity, with some individuals experiencing it intermittently while others may struggle with it continuously. Importantly, suicidal ideation may or may not lead to action and is often considered an indicator of underlying mental health issues, such as depression or anxiety. In contrast, SCS is a more immediate and intense emotional state, often triggered by a crisis, that can lead to impulsive suicidal actions.

Our hypotheses were that (a) the national health indices, namely the GHS-I and the LPH-I, would be associated with individuals’ SCI scores during the COVID-19 pandemic because individuals and governments of countries with a robust health security infrastructure were better equipped to handle the challenges posed by the pandemic, which, in turn, may have resulted in reduced concerns about contracting the virus; (b) individuals who had had COVID-19 symptoms in the past month would have higher SCI scores than those without such symptoms because previous studies found evidence for increased rates of suicide ideation in participants with COVID-19;^12^ (c) and national health indices and the presence of COVID-19 symptoms in the past month would interact, in that individuals experiencing COVID-19 symptoms would have lower SCI scores if they lived in a country with a well-developed health infrastructure than if they lived in one with a less well-developed health infrastructure.

## Method

### Sample

Between June 2020 and September 2021, 12 046 participants were recruited for the international online survey. Participants with missing values in one or more experimental variable were excluded from the data analysis, so the final sample consisted of 11 848 (98.4%) participants from 12 countries (Argentina, *n* = 476; Brazil, *n* = 2202; Canada, *n* = 94; Germany, *n* = 541; India, *n* = 306; Israel, *n* = 200; Mexico, *n* = 3390; Poland, *n* = 584; Russia, *n* = 612; South Korea, *n* = 1043; Turkey, *n* = 457; and the USA, *n* = 1943). Sociodemographic information can be found in Table 1.

**Table 1:** Sociodemographic information for the full sample and at the country level

Sociodemographic information		Total	ARG	BRA	CAN	GER	IND	ISR	KOR	MEX	POL	RUS	TUR	USA
Psychiatric diagnosis, %		22.22	32.14	29.61	39.36	13.49	6.21	23.50	5.18	14.37	26.03	12.75	26.70	39.01
Education, %														
Incomplete^a^		0.12	0.21	0.00	1.06	0.37	0.00	0.00	0.00	0.03	1.03	0.00	0.00	0.15
Non-high school^a^		3.99	0.00	0.09	4.26	12.94	0.99	0.50	0.19	1.86	44.18	1.31	13.57	0.00
High school^a^		37.05	52.52	3.59	42.55	22.74	16.68	9.00	16.68	81.74	0.00	51.47	21.23	24.24
Vocational^a^		10.98	13.03	35.20	0.00	0.00	0.00	40.50	0.00	0.00	4.80	23.20	11.16	8.34
Academic^a^		47.86	34.24	61.13	52.13	63.96	82.45	50.00	83.13	16.37	50.00	24.02	54.05	67.27
Occupation, %														
Employed^b^		56.41	31.30	47.55	52.13	63.40	43.14	50.50	55.09	64.54	47.95	34.80	66.74	67.01
Student		24.92	22.69	47.32	15.96	22.55	16.99	24.50	29.37	9.91	22.95	55.72	10.07	20.64
Retired		2.15	2.52	1.68	4.26	6.29	19.94	8.50	0.48	0.56	1.97	22.88	1.97	0.88
Unemployed		6.86	12.40	2.41	18.09	3.70	8.50	14.00	5.18	5.96	13.01	4.58	15.10	9.32
Other^b^		9.66	31.09	1.05	9.57	4.07	11.44	2.50	9.89	19.03	11.64	2.61	6.13	2.16
Living status, %														
Alone		13.92	6.93	10.85	19.15	21.55	7.19	8.08	23.59	10.86	13.06	18.53	9.65	18.43
Family/partner		75.35	86.35	82.93	63.83	67.28	87.91	86.36	71.05	80.89	74.91	61.94	85.75	58.26
Roommates		7.62	0.63	4.09	17.02	9.43	2.29	17.02	4.12	3.19	8.94	15.69	3.07	21.51
Other		3.11	6.09	2.13	0.00	1.85	2.61	0.00	1.25	5.07	3.09	3.84	1.54	1.80

ARG, Argentina; BRA, Brazil; CAN, Canada; GER, Germany; IND, India; ISR, Israel; KOR, South Korea; MEX, Mexico; POL, Poland; RUS, Russia; TUR, Turkey; USA, United States of America.

a. Education (the highest education level was recorded): incomplete, secondary education not completed; non-high school, any secondary education certificate/diploma that does not entitle the holder to study at a university; high school, general university entrance qualification; vocational, post-secondary vocational education that is typically offered outside university; academic, post-secondary academic education such as a bachelor, master or PhD.

b. Occupation: Employed includes paid, full-time and part-time employment; Other includes volunteers, work and travel, homemakers, individuals with illnesses or disabilities and any response to the questionnaire item ‘other, which: …’.

### Procedure

The present study was part of the International Suicide Prevention Assessment Research for COVID-19 collaboration. Participants were recruited online by the participating institutions in each of the above-mentioned countries via paid advertisements and postings on social media and other websites. The postings included an anonymous link to the secure online platform Qualtrics. After providing online informed consent, participants responded to a survey battery containing 250–300 items (some countries/institutes used additional questionnaires that are not part of the present analysis). The whole procedure took approximately 30–40 min to complete. Compensation was offered only in South Korea (3000 Korean won) and the USA (raffle for one of 30 US$15 gift cards). All survey batteries were translated (by forward and backward translation) into the countries’ main languages by two independent bilingual translators.

The authors assert that all procedures contributing to this work comply with the ethical standards of the relevant national and institutional committees on human experimentation and with the Helsinki Declaration of 1975, as revised in 2013.

All procedures involving human participants were approved by the relevant institutional review boards or institutional ethics committees from the affiliated institutions of all principal investigators (Argentina: no separate ethical approval was needed as there was approval from the US review board; Brazil: Ethics Committee in Research and Humans of the Universidade do Extremo Sul Catarinense, approval number 4275326; Canada: Centre for Addiction and Mental health Research Ethics Board (CAMH REB), 108-2020; Germany: Ethik-Kommission der Bayerischen Landesärztekammer, 20047; India: Institutional Ethics Committee for Observational Studies, JIP/IEC/2020/190; Israel: The College of Management Academic Studies, 0129-2020; Mexico: Comité de ética en Investigación. Instituto Nacional de Psiquiatría Ramón de la Fuente Muñiz, CEI/C/259/2020; Poland: Committee for Ethics in Scientific Research of the Institute of Psychology, Polish Academy of Sciences, # 22/VII/2020; Russia: no separate ethical approval was needed as there was approval from the US review board; South Korea: Chungbuk National University, CBNU-202007-HR-0120; Turkey: Erenkoy Mental and Nervous Diseases Training and Research Hospital’s Clinical Research Ethics Committee, 20.07.2020/29; USA: Mount Sinai Institutional Review Board, STUDY-20-00616).

### Material

The online survey collected sociodemographic information and data on current living situations. Given the unavailability of COVID-19 tests during the initial stages of the pandemic, potential illness was assessed by the following survey item: ‘The World Health Organization (WHO) describes the most common symptoms of coronavirus (COVID-19) as fever, tiredness and dry cough. In the past month, have you experienced one or more of the above symptoms?’

#### SCI

The SCI is a 49-item self-report questionnaire for estimating short-term suicide risk by asking about symptoms of SCS.^19^ Participants assessed each symptom on a 5-point Likert scale ranging from 0 (’not at all’) to 4 (’extremely’). A total score was computed by summing the item scores. The SCI has very good psychometric properties, which were tested for eight different countries (item difficulty, reliability, construct validity).^19–27^ In the present sample, reliability (Cronbach’s alpha) was excellent (0.97 ≤ α ≤ 0.98 by country).

#### Health security indices

The GHS-I originates from a collaboration between the Nuclear Threat Initiative and the Johns Hopkins University Center for Health Security. It assesses the ability of 195 countries to fight infectious disease outbreaks by using 37 indicators and 96 subindicators, which are merged to six subscales: (a) prevention; (b) detection and reporting; (c) rapid response; (d) health system; (e) commitments to improving national capacity, financing and global norms; and (f) risk environments. It also provides a total score that ranges from 0 to 100, where 100 indicates the best conditions to fight infectious disease outbreaks.^18^ The 2021 GHS-I total scores for the countries included in the present sample are shown in Table 2.

**Table 2:** Overview of Suicide Crisis Inventory (SCI) scores and other variables in the total sample and per country

Country	SCI	Gender	Age	COVID-19 symptoms in past month	Death incidence^a^	GHS-I^b^	LPH-I^b^
	mean (s.d.)	Male, %	mean (s.d.)	Yes, %	mean (s.d.)	mean (s.d.)	mean (s.d.)
Total	44.56 (36.82)	24.72	32.40 (12.26)	18.98	11.29 (11.94)	55.87 (9.19)	74.57 (3.96)
ARG	60.79 (38.55)	12.40	32.60 (11.55)	26.68	24.39 (6.43)	54.40	77.19
BRA	54.46 (39.43)	28.43	31.31 (10.90)	18.34	32.36 (7.84)	51.20	72.02
CAN	45.20 (33.07)	28.72	34.82 (9.54)	17.02	4.54 (3.40)	69.80	78.37
GER	31.70 (30.24)	24.77	40.00 (15.48)	21.44	1.19 (3.53)	65.50	81.12
IND	25.18 (30.05)	28.43	43.61 (17.96)	13.07	2.11 (0.46)	42.80	67.07
ISR	36.74 (29.88)	19.00	36.42 (17.07)	17.00	7.08 (1.82)	47.20	82.84
KOR	52.53 (34.29)	27.80	30.37 (8.21)	13.42	0.07 (0.05)	65.40	84.06
MEX	30.12 (34.06)	29.12	35.32 (12.20)	15.13	7.81 (3.03)	57.00	72.68
POL	51.32 (36.38)	13.36	32.24 (13.35)	29.45	1.81 (2.12)	55.70	75.24
RUS	40.37 (29.76)	23.69	27.21 (13.09)	25.98	3.73 (2.52)	49.10	71.64
TUR	53.78 (37.36)	40.04	33.47 (9.54)	16.63	3.94 (2.19)	50.00	75.09
USA	54.26 (33.42)	11.32	26.61 (8.41)	23.26	8.27 (4.25)	75.90	73.95

ARG, Argentina; BRA, Brazil; CAN, Canada; GER, Germany; IND, India; ISR, Israel; KOR, South Korea; MEX, Mexico; POL, Poland; RUS, Russia; TUR, Turkey; USA, United States of America.

a. Sum of COVID-19 deaths per 100 000 inhabitants in the past month.

b. GHS-I, Global Health Security Index; LPH-I, Legatum Prosperity Health Ia ndex. Scores can range between 0 and 100, where higher values indicate better health conditions for the GHS-I and LPH-I in 2021. At the country level, official scores are shown rather than means and standard deviations.

The Legatum Prosperity Index is part of an annual report of the Legatum Foundation that aims to provide indicators to compare 167 countries by using three upper domains, 12 lower pillars and 300 indicators that are thought to be central for people to have ‘the opportunity to thrive by fulfilling their unique potential and playing their part in strengthening their communities and nations’.^28^ For the purpose of the present study, we used the health pillar score (LPH-I), which estimates access to health services and maintenance of good health in countries with a score ranging from 0 (worst case) to 100 (best case) and uses 29 indicators that are merged to six subscales: (a) behavioural risk factors, (b) preventative interventions, (c) care systems, (d) mental health, (e) physical health and (f) longevity.^28,29^ The 2021 LPH-I scores from are shown in Table 2.

#### 2019 Novel Coronavirus Dataset

Some weeks after the first occurrence of COVID-19 in Wuhan (China), researchers at the John Hopkins University provided an online dashboard that illustrated the global spread of COVID-19. Thereafter, the location (country) and numbers of newly confirmed cases, deaths and recovered patients were added regularly.^30^ The open-access raw data can be downloaded via GitHub or, as in the present study, accessed through the R package coronavirus.^31^

In this study, we used the 2019 Novel Coronavirus Dataset to estimate the local severity of the COVID-19 pandemic at the time of data collection. During data collection, daily cases were affected by several dynamic variables, such as dangerousness of SARS-CoV-2 variants; availability, performance and progress of vaccination; and local strategies against COVID-19. For this reason, we used the 28-day sum of COVID-19 deaths as an estimate of the local severity, which was calculated specifically for each participant, based on the country they were in and the date of their participation in the survey.

### Data analysis

Data were analysed with R Version 4.2.1 for Windows (Vienna University of Economics and Business, Vienna, Austria; see https://cran.r-project.org/) with the packages tidyverse, nlme, coronavirus and psych.^31–34^ As a first step, raw data were prepared for data analysis, which included computation of the national 28-day sum of deaths per 100 000 inhabitants for each participant (death incidence) and *z*-transformation of non-binary predictors. Multilevel models were used because the estimation of the intraclass correlation was greater than 0.05.^35^ Multilevel models were computed by the maximum likelihood estimation. Starting with a random-intercept model, we used a stepwise approach that included the comparison of each model by likelihood ratio tests (see Table 3). Effect sizes were estimated with individual-level explained variance (*R* ^2^_1_) and country-level explained variance (*R* ^2^_2_).^36^

**Table 3:** Factors influencing the Suicide Crisis Inventory scores. Stepwise comparison of each multilevel model by using likelihood ratio tests

Model	*R* ^2^_indivual_	*R* ^2^_country_	df	χ^2^(1)	*p*
Random-intercept model (country)^a^	–	–	2	–	–
+ gender	0.015	0.078	3	180.90	<0.001
+ age	0.053	0.336	4	463.35	<0.001
+ COVID-19 symptoms	0.066	0.342	5	164.23	<0.001
+ death incidence^b^	0.070	0.484	6	60.25	<0.001
GHS-I models^c^					
+ GHS-I	0.070	0.537	7	1.33	0.249
+ GHS-I × COVID-19 symptoms	0.072	0.542	8	22.81	<0.001
LPH-I models^d^					
+ LPH-I	0.070	0.561	7	1.84	0.175
+ LPH-I × COVID-19 symptoms	0.071	0.565	8	5.48	0.019

a. Each model includes all predictors of the previous model plus (+) the predictor of the current row; LPH-I models do not include the additional predictors of the GHS-I models.

b. Death incidence, countries’ sum of COVID-19 deaths per 100 000 inhabitants in the past month.

c. GHS-I, Global Health Security Index.

d. LPH-I, Legatum Prosperity Health Index.

## Results

Descriptive statistics of the variables used for multilevel analyses are shown in Table 2. One-way analyses of variance were computed to test differences between countries in the SCI score and all non-binary predictors except the GHS-I and LPH-I. They indicated significant differences (*p* < 0.001) between countries in the SCI score (*F* [11, 11 836] = 119.68, *η^2^* = 0.100) and the non-binary predictors age (*F* = 129.51, *η^2^* = 0.107) and death incidence (*F* = 6546.90, *η^2^* = 0.859). In addition, chi-square tests showed significant differences (*p* < 0.001) between countries regarding gender (*χ* ^2^ [11] = 463.23, *V* = 0.198) and experiencing COVID-19 symptoms in the past month (*χ* ^2^ = 168.28, *V* = 0.119).

Multilevel modelling included a comparison of ten different models (Table 3). The final models (Table 4) showed that age, gender, having COVID-19 symptoms in the past month and the COVID-19 death incidence of the country in which participants lived influenced SCS severity. Specifically, for each one-unit increase (= 1 standard deviation) in age, the SCI score decreased by 6 points. Thus, severity of SCS decreased with age. The results further showed that women scored 9 points higher than men. In addition, participants with COVID-19 symptoms in the past month scored 10 points higher than those who did not have COVID-19 symptoms, and a one-unit increase in a country’s COVID-19 death incidence at the time of survey participation led to a 6-point increase in the SCI score. In brief, severity of SCS was higher in younger participants, women, participants with COVID-19 symptoms and participants living in countries with a higher COVID-19 death incidence.

**Table 4:** Factors influencing the Suicide Crisis Inventory scores. Final models for the Global Health Security Index (GHS-I) and Legatum Prosperity Health Index (LPH-I)

Factors	GHS-I model		LPH-I model	
	*b* (s.e. *b*)	95% CI	*b* (s.e. *b*)	95% CI
Intercept	38.69 (2.34)***	34.10, 43.28	37.34 (2.32)***	32.80, 41.88
Gender^a^	9.32 (0.73)***	7.88, 10.76	9.33 (0.73)***	7.89, 10.77
Age	−6.66 (0.33)***	−7.31, –6.01	−6.72 (0.33)***	−7.37, –6.08
COVID-19 symptoms^b^	10.32 (0.80)***	8.75, 11.88	10.12 (0.80)***	8.56, 11.69
Death incidence^c^	6.31 (0.79)***	4.76, 7.86	6.26 (0.79)***	4.72, 7.81
GHS-I	3.24 (2.13)	−1.51, 7.99		
GHS-I × COVID-19 symptoms	−3.65 (0.76)***	−5.15, –2.15		
LPH-I			2.86 (1.80)	−1,14, 6.86
LPH-I × COVID-19 symptoms			−2.00 (0.85)*	−3.67, –0.33

a. Gender: 0 = male, 1 = female.

b. COVID 19 symptoms: 0 = false, 1 = true.

c. Death incidence = countries’ sum of COVID-19 deaths per 100 000 inhabitants in the past month.

***p < 0.001, **p < 0.01, *p < 0.05; non-binary predictors were *z*-transformed.

The results did not reveal a significant main effect of the health security system predictors on the SCI score of participants from the various countries. However, we observed a significant interaction between the predictors for the health security systems and COVID-19 symptoms in that the SCI score was reduced (by 3.65 if the GHS-I was considered and by 2 if the LPH-I was considered) if the indices of the health scores increased by one unit and the participant had had symptoms of COVID-19 in the past month. Thus, the severity of SCS in participants with COVID-19 symptoms was lower in countries with a better-resourced healthcare system.

## Discussion

The present study analysed country-level differences in SCS severity during the COVID-19 pandemic. For this purpose, SCS was estimated from SCI scores. The results showed significant differences in countries’ mean SCI scores. The corresponding effect size was moderate to strong, that is, country-level differences accounted for 10.0% of the variance in SCI scores. The impact of different predictors on individual- and country-level variance was examined by multilevel model analyses. Overall, these analyses revealed that age, gender, having COVID-19 symptoms, a country’s COVID-19 death incidence and interactions between health security scores and having COVID-19 symptoms significantly predicted SCI scores. The final models that included these predictors and interactions accounted for around 55% of the SCI variance on a country level and for around 7% of the variance on an individual level.

The GHS-I and LPH-I both assess the quality of countries’ health security system and provide important information about countries’ ability to deal with severe health challenges, such as the COVID-19 pandemic. We assumed that these indices would have been associated with individuals’ SCI scores because the more successful a country was in dealing with the pandemic, the fewer individuals would worry about their lives. However, the results of the current study did not support the assumption of a main effect of health indices on the SCI: neither the GHS-I nor the LPH-I significantly predicted SCI scores. Consistent with our hypotheses, participants with COVID-19 symptoms reported higher SCS severity, while those living in countries with an advanced healthcare infrastructure reported lower SCI scores after contracting COVID-19. This reflects their perception of being well supported and cared for and underscore the significant influence of national health policies on the population’s mental well-being.

Examining the control variables reveals the following pattern: the number of COVID-19 deaths (at the time participants completed the survey and in their respective countries) served as an indicator of the perceived threat level. Consistent with this, SCS symptoms were more pronounced as the number of deaths increased. In addition, female gender and younger age were associated with higher SCI scores. This finding aligns with previous studies on suicide ideation during the COVID-19 pandemic^13,14^ and is supported by epidemiological evidence. While men are three times more likely to die by suicide,^37^ women have higher lifetime prevalence rates of depression and suicide attempts compared with men.^38,39^ Similarly, higher suicide rates in adolescents and young adults are well documented. Young people, in particular, are in a critical phase of emotional and psychological development, which can make them more susceptible to stress, identity crises and social insecurities, factors that may increase the risk of a suicidal crisis.^40^

Higher age explained variance on the country level to a high extent, suggesting that differences in the mean age in individual countries must be considered when interpreting SCI scores. For example, participants in the USA had the lowest mean age (26.61 years) and third highest mean SCI score (54.26), whereas participants in India had the highest mean age (43.61 years) and the lowest mean SCI score (25.18).

### Limitations

This study has strengths, such as a large sample size with mostly large national subsamples; however, it also has some limitations. As a convenience sample that was recruited mainly via social media, a self-selection bias can be expected. In most countries, the majority of participants were female and highly educated, which challenges the generalisability of our findings. In addition, differences in employment rates across countries may have influenced the observed effects.

In the present study, participants were asked to self-report whether they had experienced COVID-19 symptoms in the past month. This presents a limitation, as the assessment is subjective. Participants might mistakenly attribute symptoms such as coughing, sore throat or fatigue to COVID-19, even though these symptoms can also result from other infections, the flu or stress-related exhaustion. As a result, the findings may be skewed, since it does not confirm whether the symptoms were actually caused by COVID-19.

The COVID-19 pandemic was very dynamic, meaning that it consisted of several waves that differed in terms of disease severity, the spread of SARS-CoV-2 and healthcare utilisation. The international online survey included data from June 2020 to September 2021. In most countries, the survey took place in the second half of 2020; however, data were not available for every country for the same months. Taken together, these factors make it very likely that the pandemic was perceived differently by participants in different countries because of differences in the status of COVID-19 and the timing of participation. To reduce this problem, we used the 28-day sum of COVID-19 deaths in the multilevel models as an estimate of pandemic severity. However, although the 28-day sum of COVID-19 deaths is considered to be less influenced by factors such as the severity of COVID-19 symptoms, vaccination rates and national strategies in combating the virus compared with the 28-day sum of newly confirmed cases, it may still have been affected by variations in local practises for attributing deaths to COVID-19. For example, national guidelines in the USA allowed COVID-19 to be attributed as the cause of death without a positive COVID-19 test if the patient had typical COVID-19 symptoms before they died.^41^ Another significant limitation was the lack of detailed information regarding the characteristics of each country’s health systems.

The study shows that during a pandemic, it is crucial for health systems to provide adequate resources to meet the increased needs of infected individuals and of residents of countries with high death incidences. The necessary measures include promoting accessible and affordable health services, expanding telehealth options and implementing community-based interventions. Advanced health systems can play a vital role in mitigating the negative impact of a national or international health crisis on suicidal ideation and behaviour and supporting individuals’ overall well-being.

## Data Availability

The data and analytic code that support the findings of this study are available from the corresponding author, F.B., upon reasonable request.
